# Adding exogenous biglycan or decorin improves tendon formation for equine peritenon and tendon proper cells in vitro

**DOI:** 10.1186/s12891-020-03650-2

**Published:** 2020-09-23

**Authors:** Monica Y. Pechanec, Tannah N. Boyd, Keith Baar, Michael J. Mienaltowski

**Affiliations:** 1 Department of Animal Science, University of California Davis, 2251 Meyer Hall, One Shields Ave, Davis, CA 95616 USA; 2 Department of Neurobiology, Physiology, and Behavior, University of California Davis, 195 Briggs Hall, One Shields Ave, Davis, CA 95616 USA; 3 Department of Physiology and Membrane Biology, University of California Davis School of Medicine, 195 Briggs Hall, One Shields Ave, Davis, CA 95616 USA

**Keywords:** Equine, Tendon, Peritenon, Biglycan, Decorin, Three-dimensional construct

## Abstract

**Background:**

Tendon injuries amount to one of the leading causes of career-ending injuries in horses due to the inability for tendon to completely repair and the high reinjury potential. As a result, novel therapeutics are necessary to improve repair with the goal of decreasing leg lameness and potential reinjury. Small leucine-rich repeat proteoglycans (SLRPs), a class of regulatory molecules responsible for collagen organization and maturation, may be one such therapeutic to improve tendon repair. Before SLRP supplementation can occur in vivo, proper evaluation of the effect of these molecules in vitro needs to be assessed. The objective of this study was to evaluate the effectiveness of purified bovine biglycan or decorin on tendon proper and peritenon cell populations in three-dimensional tendon constructs.

**Methods:**

Equine tendon proper or peritenon cell seeded fibrin three-dimensional constructs were supplemented with biglycan or decorin at two concentrations (5 nM or 25 nM). The functionality and ultrastructural morphology of the constructs were assessed using biomechanics, collagen content analysis, transmission electron microscopy (TEM), and gene expression by real time – quantitative polymerase chain reaction (RT-qPCR).

**Results:**

SLRP supplementation affected both tendon proper and peritenon cells-seeded constructs. With additional SLRPs, material and tensile properties of constructs strengthened, though ultrastructural analyses indicated production of similar-sized or smaller fibrils. Overall expression of tendon markers was bolstered more in peritenon cells supplemented with either SLRP, while supplementation of SLRPs to TP cell-derived constructs demonstrated fewer changes in tendon and extracellular matrix markers. Moreover, relative to non-supplemented tendon proper cell-seeded constructs, SLRP supplementation of the peritenon cells showed increases in mechanical strength, material properties, and collagen content.

**Conclusions:**

The SLRP-supplemented peritenon cells produced constructs with greater mechanical and material properties than tendon proper seeded constructs, as well as increased expression of matrix assembly molecules. These findings provide evidence that SLRPs should be further investigated for their potential to improve tendon formation in engineered grafts or post-injury.

## Background

Tendinopathies like those of the superficial digital flexor tendon (SDFT) result in major leg lameness and are debilitating for horses of all disciplines [1, 2]. For both acute and chronic tendinopathies, like those of the SDFT, a closer look at the pathology associated with tendon injury oftentimes implicates alterations in extracellular matrix (ECM) regulators of collagen fibrillogenesis and organization [3]. Alterations in the expression of ECM regulators lead to changes in biomechanical properties that impact the strength and stability of these energy storing tendons [4]. Due to this, novel therapeutics are necessary since complete repair is unlikely and further injury is a major concern [2, 3].

Small leucine-rich repeat proteoglycans (SLRPs) are a class of regulatory molecules that are essential for collagen organization in tendon development, maturation, and repair [5]. The contributions of SLRPs have been particularly well-characterized in tendons [6–12]. Besides directly affecting collagen fibrillogenesis, SLRPs like biglycan (BGN) and decorin (DCN) play roles in determining how tissue niche impacts cell biology, including: 1) the differentiation status of tendon progenitors in health and pathology [13]; 2) inflammatory regulation as Damage Associated Molecular Pattern proteins interacting with Toll-like receptors [14]; 3) recruitment of cells to sites of tissue repair or regeneration [15]; and 4) sequestration of growth factors essential for generation and maintenance of the tendon phenotype [13]. Previous work has demonstrated that the absence of these SLRPs dramatically affects tendon repair outcomes with BGN essential early in repair and DCN crucial later in tendon repair [7, 9]. Interestingly, expression of *BGN* and *DCN* in mature animals decreases after an injury and never recovers to the level seen during development and maturation [11], suggesting that low BGN and/or DCN may contribute to the impaired injury response.

After a mature tendon is injured, repair occurs as a result of extrinsic and intrinsic influences. Leukocytes and fibroblasts migrate into the lesion early in repair [15]. Post-injury, these fibroblasts originate from the extrinsic paratenon and have demonstrated distinct differences in marker expression and tenogenic potential as compared to the tendon proper fibroblast cell population [16–20]. Thus, when considering therapeutic interventions for tendon repair, both cell populations should be included since the role of each cell type remains unresolved.

Recognizing the value of BGN and DCN in tendon development and maturation and their subsequent decline at the time of repair, we hypothesize that addition of BGN or DCN to the tendon matrix would improve tendon formation. To test this hypothesis, equine tendon proper (TP) and peritenon (PERI) cells were seeded in an in vitro fibrin-based three-dimensional tendon construct model in which the gel contained two differing amounts of either exogenous bovine purified BGN or exogenous bovine purified DCN. The effects of the exogenous BGN or DCN on biomechanics, electron microscopic ultrastructure, collagen content, and gene expression were determined.

## Methods

### Tendon harvest and cell isolation

Equine superficial digital flexor tendon (SDFT) cells were harvested from five horses of various breeds (ages 8–15 years) with approval from the University of California Davis Institutional Animal Care and Use Committee. All horses were property of the University of California Davis and were assessed as healthy with no known tendinopathies and were euthanized by intravenous injection of euthanasia solution (pentobarbital sodium and phenytoin sodium) for reasons unrelated to the study. After euthanasia, 2.5 cm of forelimb SDFT was harvested per horse approximately 10–15 cm proximal of the forelimb fetlock. Tendons were transported to the lab in Dulbecco’s Phosphate Buffer Solution (DPBS, Life Technologies, Benicia, CA, USA) containing 1% antibiotic/antimycotic (10,000 units/mL penicillin, 10,000 μg/mL streptomycin, and 25 μg/mL amphotericin B, Life Technologies). For each horse, tissue from the tendon proper and peritenon regions were isolated while submerged in DPBS containing 1% antibiotic/antimycotic. Under a dissecting microscope and sterile conditions, the peritenon region was isolated by removing the paratenon and 1 mm of the epitenon region using forceps and sterile scalpel blades. The tendon proper region of the tendon was isolated by removing a 2 mm square the length of the sample of the tendon core [5, 18, 20]. Separated regions were then used for digestion to isolate the different cell populations for each horse. Enzymatic digestion followed previous protocols using 0.3% type-I collagenase (CLS-1, Worthington, Lakewood, New Jersey, USA) and 0.4% Dispase II (Roche, Basel, CH) in Hanks Balanced Salt Solution (HBSS, Gibco, Benicia, CA, USA) with enzymatic inactivation after agitation in standard tenocyte media (alpha-MEM, 10% fetal bovine serum, 2 mM L-glutamine, and 1% antibiotic/antimycotic) [20, 21]. Cells from each region for each horse were plated in T75 flasks and expanded in standard tenocyte media. Cells from each region for each horse were passaged before being cryopreserved in 10% dimethyl-sulfoxide (DMSO) solution in standard tenocyte culture media under liquid nitrogen after reaching P2.

To make constructs, frozen vials of peritenon and tendon-proper cells were thawed and seeded as P3 in T75 flasks at 6666 cells per cm^2^ and grown to 85% confluency in normal tenocyte media. Tear-drop shaped brushite anchors (100 mM citric acid and 3.5 M orthophosphoric acid added to dense β-tricalcium phosphate mixture (β-TCP; Plasma Biotal Limited, Derbyshire, UK) in a 1 mL per 1 g ratio) were pinned 1 cm apart in 35 mm tissue culture treated dishes cured with 3 mL of Sylgard (184 Silicone Elastomer Kit, Dow Corning, Midland, MI) [21–23]. For each horse sample and treatment, a minimum of 3 constructs were used for biomechanics and subsequent collagen analysis, 1 for real time quantitative polymerase chain reaction (RT-qPCR), and 1 for transmission electron microscopy (TEM). Bovine biglycan (bBGN) (Sigma-Aldrich) or bovine decorin (bDCN) (Sigma-Aldrich) was supplemented into the fibrin gel mixture at high (25 nM) or low (5 nM) concentrations – doses that were previously investigated with cultured myotubules, cardiomyocytes, and type I collagen gels [24–26]. Therefore, at least 50 constructs were made for each horse in order to provide a minimum of 5 technical replicates for the control, high and low bBGN, and high and low bDCN conditions for both the peritenon and tendon proper cells. To make the tendons, cells were combined with the fibrinogen-thrombin matrix gel (681 μl cell suspension with supplementation or control media, 286 μl of 20 mg/mL fibrinogen, and 29 μl of 200 U/mL thrombin to get 998 μl total gel volume) at 300,000 cells per construct and seeded in a spread method around the anchors [21]. The suspension was allowed to gel for 15 min before adding tenocyte standard media supplemented with 200 uM ascorbic-2-phosphate into the wells [20]. Constructs were maintained at 37 °C in 5% CO_2_ for 14 days with media changes every 2–3 days.

### Biomechanical testing

At day 14, length and width of a minimum of 3 constructs for each treatment was determined using digital calipers before being loaded into a horizontal uniaxial tensile testing machine within a saline bath [27–29]. Samples were tested to failure without preconditioning at a constant displacement rate of 0.4 mm/s [30]. LabVIEW (National Instruments, Austin, TX) software recorded the resulting force measurements and the load-deformation curve was used to determine the maximal tensile load (MTL) of the construct. The load and deformation values were normalized to the cross-sectional area (CSA) and initial construct length, respectively, to calculate stress and strain. The ultimate tensile stress (UTS) was recorded as the highest stress value before failure, whereas Young’s modulus was determined by calculating the slope of the linear portion of the stress-strain curve.

### Collagen content

Following biomechanical testing, constructs were removed from anchors, patted dry, and placed on glass to dehydrate at 120 °C for 20 mins. Dried constructs were weighed and either stored in individual tubes until necessary or immediately processed for hydroxyproline analysis. Analysis followed a previously described protocol using 6 N hydrochloric acid at 120 °C for 2 h for hydrolysis, followed by 1.5 h to evaporate the hydrochloric acid. Hydroxyproline buffer (3.3% citric acid, 2.3% sodium hydroxide, 0.8% acetic acid in water, pH 6.0–6.5) was used to resuspend the pellets and resulting solution was stored in − 20 °C until further processing [27]. Stock samples were diluted to 9:1 or 4:1 hydroxyproline buffer:stock sample to allow for more accurate colorimetric detection. Chloramine-T (14.1 mg/mL) and aldehyde perchlorate solution were added in a step-wise fashion to each diluted sample before heating, cooling, and reading the samples and standards in a UV spectrophotometer at 550 nm [31–34].

### Transmission Electron microscopy

At day 14, constructs were rinsed with phosphate buffer solution (PBS) and fixed at length by complete immersion in Karnovsky’s fixative for 2 h at 4 °C then stored in transport solution for up to 1 week before embedding. Further processing of constructs for TEM followed previously described protocols [10, 18, 29–31, 35, 36]. Briefly, fresh epoxy resin was used to embed constructs cut in thirds cross-sectionally and polymerized for 12 h at 60 °C (EMBed – 812, Electron Microscopy Sciences, Hatfield, PA, USA). Blocks sectioned at 70 nm by ultramicrotome were post-stained with 2% aqueous uranyl acetate and 1% phosphotungstic acid, pH 3.2 [20]. Images were taken at 80 kV using a FEI C120 transmission electron microscope (FEI Co, Hillsboro, OR) with a Gatan Orius CC Digital camera (Gatan Inc., Pleasanton, CA). All images used for fibril diameter analysis, fibril density, collagen organization, and structure were taken at 33,000x. Fibril diameter distribution was visualized using ImageJ software (National Institutes of Health, Bethesda, MD) and means were calculated from 5 images per testing group within each biological sample with no more than 100 fibrils per image counted for a total of 500 fibril diameters per biological sample. Fibril density and fibrils per area of extracellular matrix (ECM) per image were calculated using the same 5 images as the fibril diameters.

### Total RNA isolation and real time quantitative PCR (RT-qPCR)

At day 14, constructs were snap frozen in liquid nitrogen and stored at − 80 °C until further processed. Homogenization of the tendon constructs was done using a BioSpec Tissue-Tearor and total RNA isolation was performed using the RNeasy Plus Micro Kit (QIAGEN, Valencia, CA) including a RNase-free DNase treatment (QIAGEN, Valencia, CA). Reverse transcription was performed on 500 ng total RNA using a High Capacity cDNA Reverse Transcription Kit (Life Technologies). Genes assessed included tenogenic differentiation (*MKX*, *FMOD*), ECM assembly (*BGN*, *DCN*, *COL1A1*, *LOX*), or perivascular (*CSPG4*) markers [7, 18, 20, 37, 38]. *POLR2A* was used as the housekeeping gene [19, 37, 39]. Taqman primers were designed from equine gene structure annotation (NCBI Equicab 3.0) using Primer3 or from predesigned primers (Life Technologies) (Table S-1) [40, 41]. For RT-qPCR analysis, 1 ul of cDNA template was combined with Taqman Master Mix (no UNG) (Life Technologies) and equine specific primers for a reaction volume of 20 ul in a StepOnePlus Real-Time PCR System (Applied Biosystems, Foster City, CA) [19]. Each sample of amplified cDNA was analyzed in duplicate for each gene with gene specific efficiencies calculated using LinRegPCR v 7.5 software [7, 18, 19]. The relative quantity ratios formula was used to calculate the relative quantity of mRNA for each gene [42, 43].

### Statistics

GraphPad Prism (GraphPad Software, Inc., San Diego, CA) was used for all statistical analyses. Mean values for technical replicates within biological replicates were calculated before differences of the mean values were compared within testing categories to avoid pseudoreplicates. Statistical analyses were all performed using nonparametric Wilcoxon signed-rank tests in which each treatment (5 nM bBGN, 25 nM bBGN, 5 nM bDCN, 25 nM bDCN) was compared to its corresponding control with a one-sided test applied [44–46]. The H_0_ was that the addition of SLRPs to the constructs would lead to no improvement or a decline in tenogenic features; the H_a_ was that the addition of SLRPs would improve tenogenic properties, or promote tendon formation, as described in several studies: (1) increased UTS, Young’s modulus, and MTL; (2) increased collagen content; (3) increases in collagen fibril numbers with increases in fibril diameters; (4) increased relative expression of tendon markers *SCX* and *MKX*, (5) increased expression of ECM assembly markers; and (6) decreased expression of perivascular markers [6–10, 12, 17–22, 27]. Significance level was set at *p* ≤ 0.05. Given the limited “n,” wherever analyses approached significance with *p* = 0.0625 (i.e., measurement ranks switched within one pair of the samples), it was noted in the results.

## Results

### Biomechanics and collagen content

**Fig. 1:**
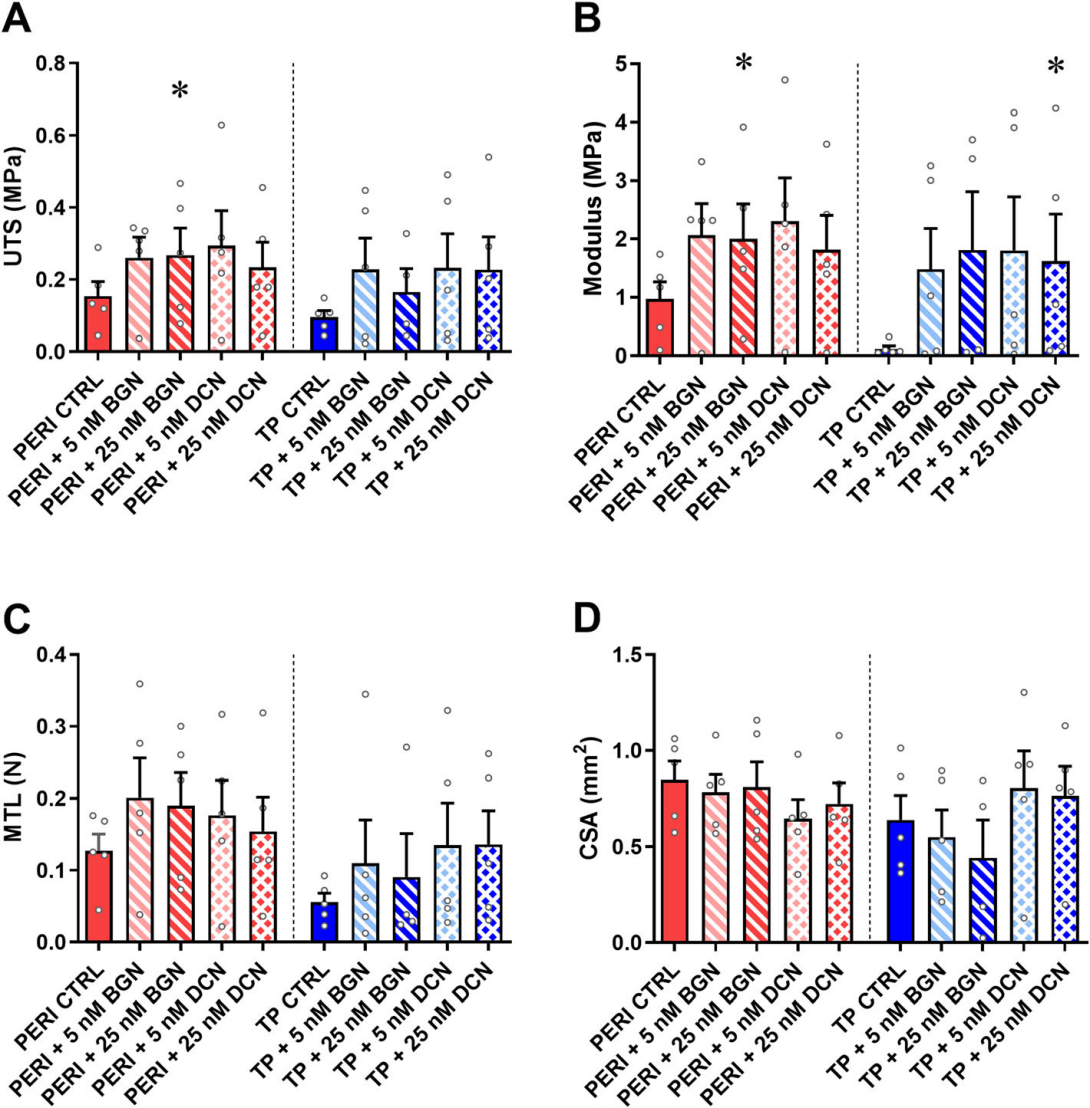
Biomechanical properties for 5 and 25 nM purified bBGN or bDCN supplementation. **a** Ultimate tensile strength (UTS), **b** Young’s modulus, and **c** Maximum tensile load (MTL) were measured across five biological replicates and plotted as mean + SEM. TP: tendon proper cells; PERI: peritenon cells; CTRL: no bBGN or bDCN supplementation. Significance is based on one-sided nonparametric Wilcoxon signed-rank tests predicting improvement: *, significant as *p* ≤ 0.05, relative to the respective TP or PERI control; *n* = 5. Outliers detected by the Grubbs’ test in technical replicates (UTS, 4; Young’s modulus, 3; MTL, 2; CSA, 1; *p* < 0.05) were removed

Constructs contracted successfully and were assessed at 14 days post seeding with cells aligned unilaterally and no qualitative differences seen between peritenon and tendon proper cells upon gross microscopic examination. The supplementation of SLRPs bBGN and bDCN improved biomechanics in many instances (Fig. 1). Peritenon cells supplemented with 25 nM bBGN showed significantly increases in UTS (*p* = 0.0313) and Young’s modulus (*p* = 0.0313). Increases in UTS approached significance (*p* = 0.0625) when PERI cells were supplemented with 5 nM or 25 nM bDCN. Increases in Young’s modulus approached significance (*p* = 0.0625) when PERI cells were supplemented with 5 nM bBGN, 5 nM bDCN, or 25 nM bDCN. For tendon proper cells, Young’s modulus significantly increased for constructs supplemented with 25 nM bDCN (*p* = 0.0313). Otherwise, for TP cells, increases in Young’s modulus approached significance (*p* = 0.0625) when supplemented with 5 nM bBGN or 5 nM bDCN. No significant increases in CSA were seen when TP or PERI cells were supplemented with bBGN or bDCN.

SLRP-treated PERI cell constructs were compared to TP cell control constructs in regard to biomechanics to discern if SLRP-supplemented PERI cells created biomechanically superior constructs (Fig. S-1). UTS was significantly greater for PERI constructs supplemented with 25 nM bBGN. Relative to TP cell control constructs, Young’s modulus was significantly greater for PERI cells supplemented with 25 nM bBGN or 5 nM bDCN, and MTL was greater for PERI cell constructs receiving 5 nM bBGN or 25 nM bDCN.

**Fig. 2:**
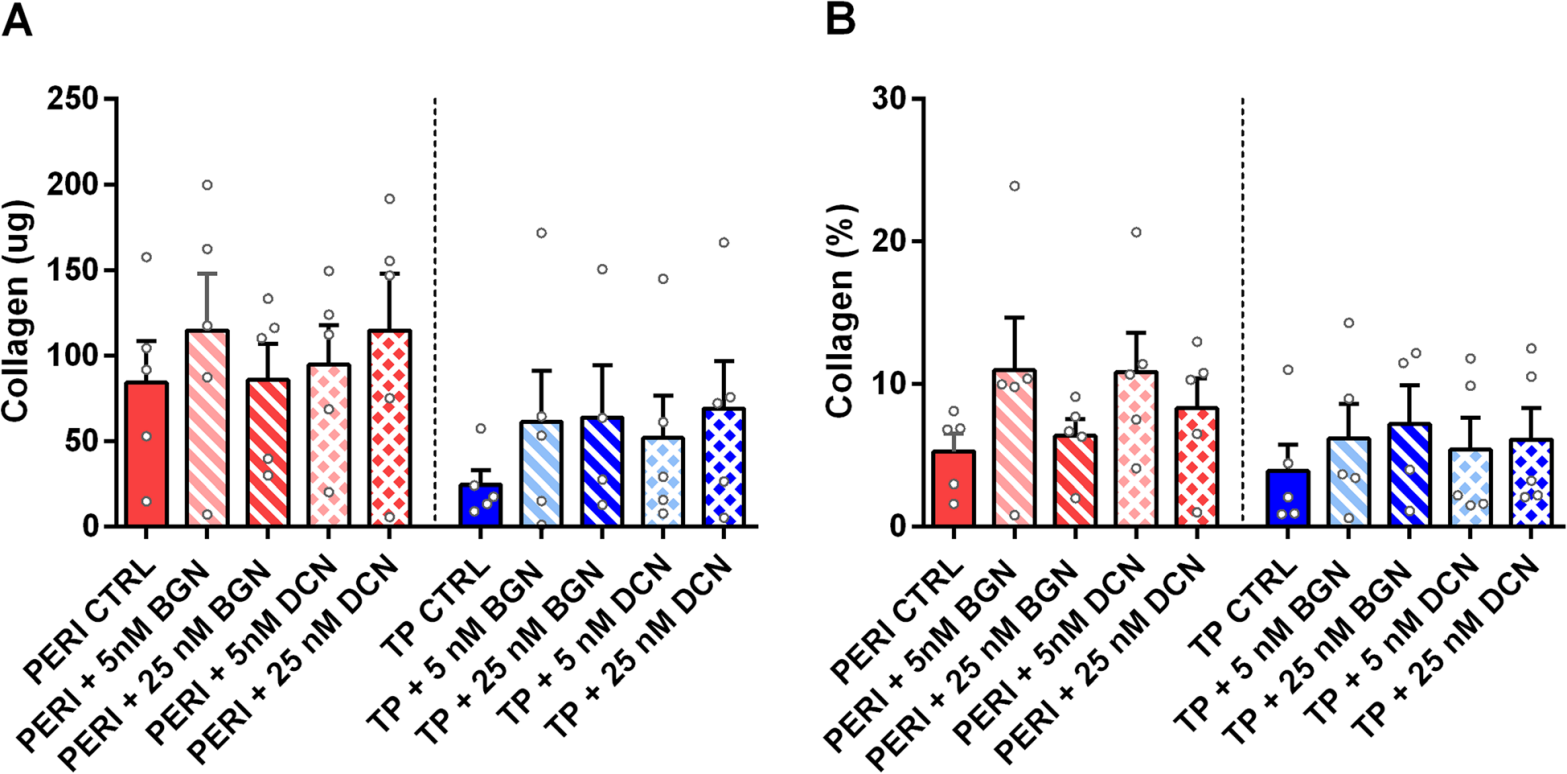
Collagen content for 5 and 25 nM purified bBGN or bDCN supplementation. Collagen analysis for (**a**) collagen content and (**b**) collagen fraction by dry mass in the tissues; plotted as mean + SEM. TP: tendon proper cells; PERI: peritenon cells; CTRL: no bBGN or bDCN supplementation. Significance is based on one-sided nonparametric Wilcoxon signed-rank tests predicting improvement: *, significant as *p* ≤ 0.05, relative to the respective TP or PERI control; *n* = 5

Increases in collagen content approached significance (*p* = 0.0625) in TP and PERI constructs supplemented with 25 nM bDCN (Fig. 2a). For collagen content as a fraction of dry mass (%), neither tendon proper nor peritenon cells showed improvement with any supplementation (Fig. 2b). Likewise, when comparing SLRP-treated PERI cell constructs with TP cell control constructs, supplementation with 25 nM bBGN or 5 nM bDCN increased collagen content (Fig. S-2A), yet not as a percentage of dry mass (Fig. S-2B).

### Transmission Electron microscopy

**Fig. 3:**
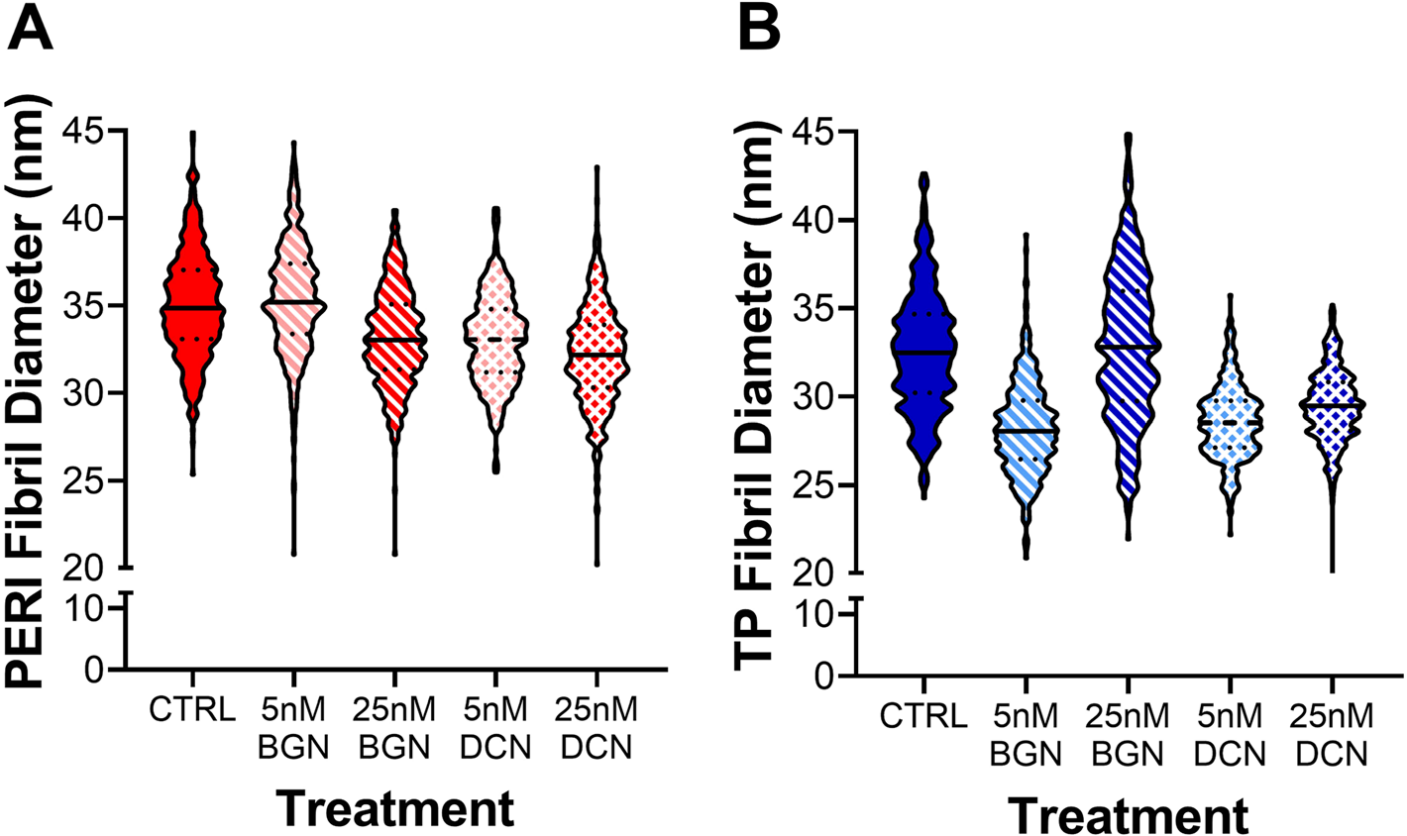
Fibril diameter analysis for samples supplemented with 5 or 25 nM bovine BGN or bovine DCN. Fibril diameter distributions are given as violin plots for constructs seeded with (**a**) peritenon (PERI) and (**b**) tendon proper (TP) cells, *n* = 5

**Fig. 4:**
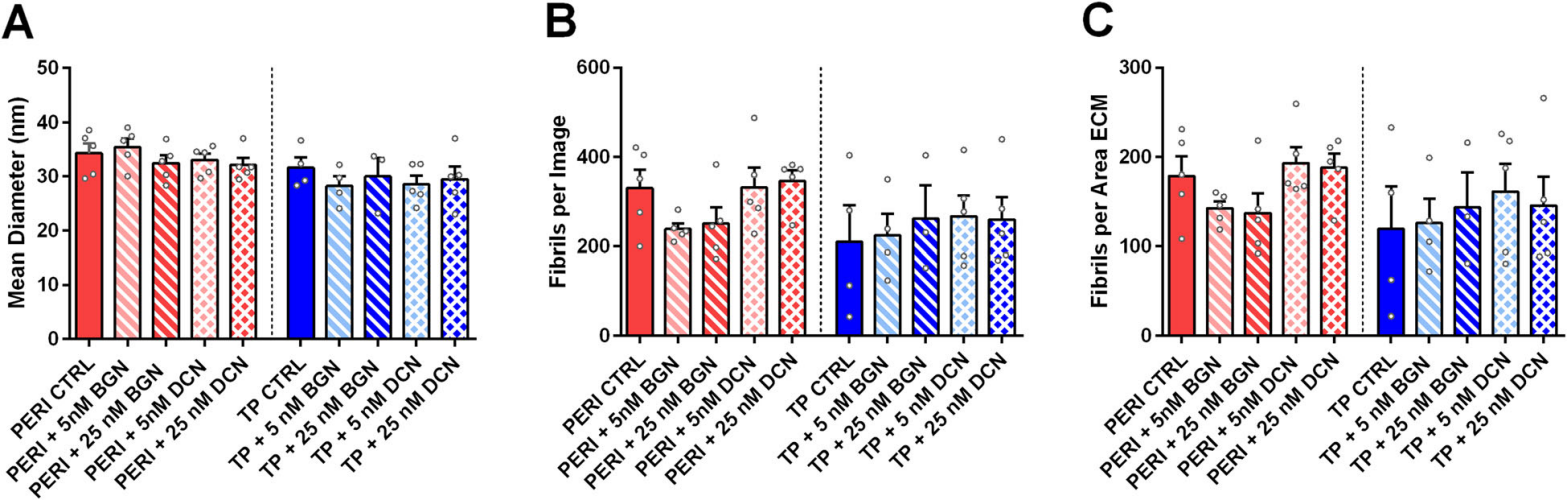
Fibril quantity analysis by mean diameter, density, and fibrils per area of extracellular matrix. **a** Mean fibril diameter, **b** Fibril number per image, and **c** fibrils per area of ECM per image were counted for all treatments; plotted as mean + SEM. TP: tendon proper cells; PERI: peritenon cells; CTRL: no bBGN or bDCN supplementation; ECM: extracellular matrix. Significance is based on one-sided nonparametric Wilcoxon signed-rank tests predicting improvement: *, significant as *p* ≤ 0.05, relative to the respective TP or PERI control; *n* = 5

TEM cross-sections were used to analyze collagen fibril diameters (Fig. S-3). Fibril analyses showed slight shifts towards smaller fibrils for PERI cells in constructs supplemented with 25 nM bBGN, 5 nM bDCN, and 25 nM bDCN, as well as for TP cells in constructs supplemented with 5 nM bBGN, 5 nM bDCN, and 25 nM bDCN (Fig. 3). A bimodal distribution can be seen for TP cell-derived constructs with 25 nM bBGN supplementation. When comparing mean fibril diameter (nm) of SLRP-supplemented constructs with their respective controls, though not statistically significant, mean diameters of PERI constructs were roughly the same size, and mean diameters of TP constructs were slightly smaller or the same size. For normalized fibrils per area of extracellular area, no significant differences were found (Fig. 4).

To understand how SLRP-treated PERI cell constructs compared to TP cell control constructs, fibril diameter distribution analyses demonstrated relative shift towards larger fibrils (Fig. S-4) and trends of more fibrils with more fibrils per area of ECM (Fig. S-5) when supplemented with bBGN and bDCN.

### Gene expression

**Fig. 5:**
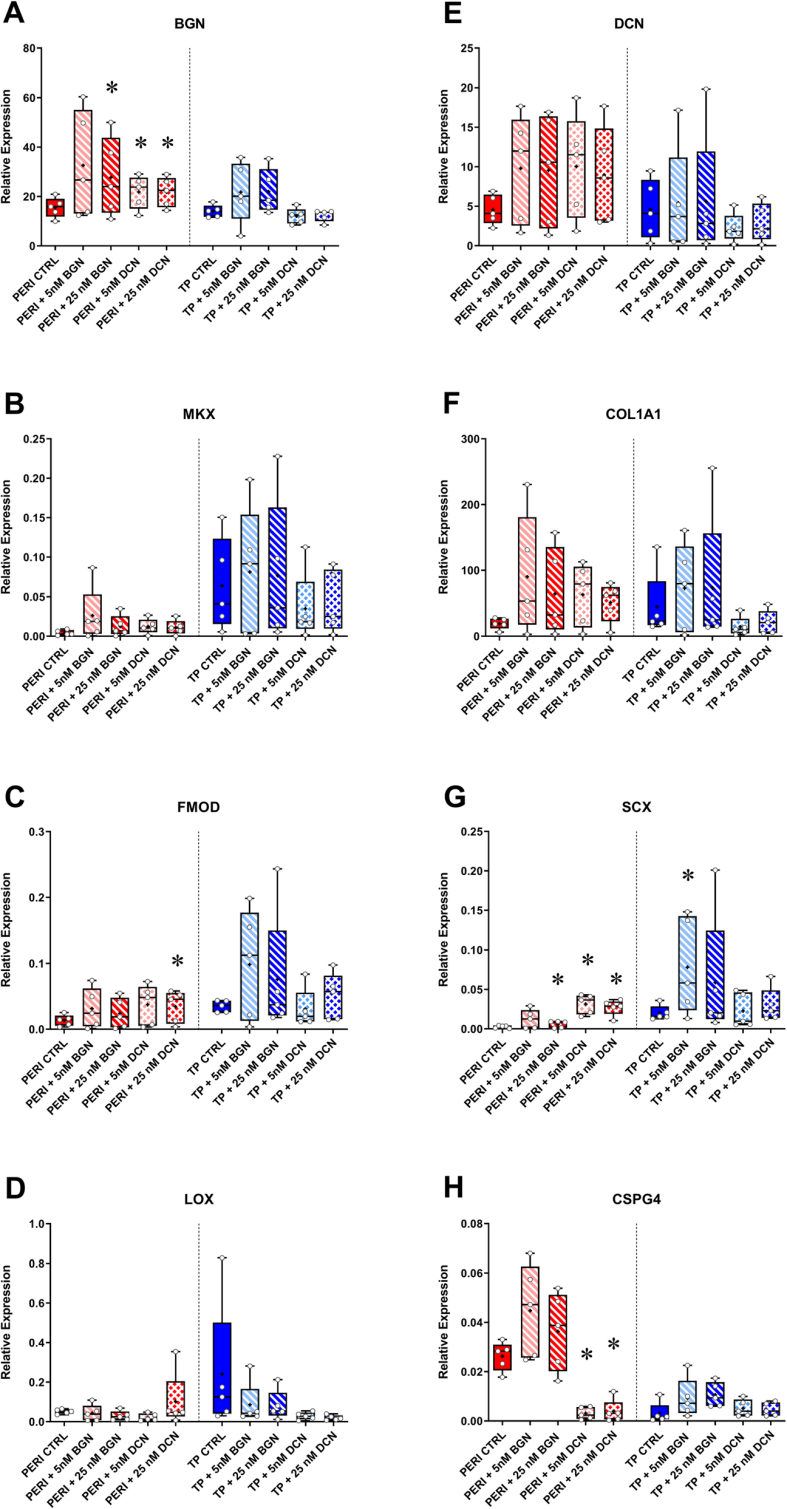
RT-qPCR analysis of perivascular and tenogenic markers. For each gene, the gene expression was plotted against the housekeeping gene *POLR2A*. TP: tendon proper cells; PERI: peritenon cells; CTRL: no bBGN or bDCN supplementation. Expression is plotted in a box and whisker plot with “+” representing the mean, box representing first-third quartile, line representing median, and whisker representing range. Significance is based on one-sided nonparametric Wilcoxon signed-rank tests predicting improvement: *, significant as *p* ≤ 0.05, relative to the respective TP or PERI control; *n* = 5

Gene expression analyses informed how SLRP supplementation within the constructs affected TP and PERI cell tenogenic properties (Fig. 5). When PERI cells in constructs were supplemented with 5 nM bBGN, increased expression of *BGN*, *SCX*, and *COL1A1* approached significance (*p* = 0.0625); however, expression of *CSPG4* was elevated. PERI cells supplemented with 25 nM bBGN had increased *BGN* and *SCX* expression (*p* = 0.0313). Supplementation of 5 nM bDCN led to increased expression (*p* = 0.0313) of *BGN* and *SCX* with an increase in *COL1A1* approaching significance (*p* = 0.0625). Moreover, expression decreased for *CSPG4* when PERI cells were supplemented with 5 nM DCN (*p* = 0.0313). PERI cells supplemented with 25 nM bDCN showed increased expression of *BGN*, *FMOD,* and *SCX* (*p* = 0.0313 for each), and decreased expression of *CSPG4* (*p* = 0.0313). Additionally, when PERI cell constructs were supplemented 25 nM bDCN, increases in expression of *MKX*, *DCN*, and *COL1A1* approached significance (*p* = 0.0625).

For the markers tested, overall fewer significant changes were seen in gene expression demonstrating improvements in tenogenesis for the tendon proper cell-derived constructs. TP cell constructs supplemented with 5 nM bBGN only demonstrated increased expression of *SCX* (*p* = 0.0313); supplementation of 25 nM bBGN led to an increase in *BGN* expression that only approached significance (*p* = 0.0625). No significant tenogenic improvements in expression were seen with supplementation of 5 nM bDCN or 25 nM bDCN.

When SLRP-treated PERI cell constructs were compared to TP cell control constructs, bBGN and bDCN PERI cell-derived constructs had similar matrix assembly marker expression levels as TP cell control constructs (Fig. S-6).

## Discussion

Embedding small leucine-rich proteoglycans within a fibrin gel affected features of the engineered tendons. When considering gene expression, the supplementation of either exogenous biglycan or decorin had a greater effect on the tenogenic capacity of the equine peritenon cells than they did on tendon proper cells. Yet, biomechanical properties were bolstered by supplementation of SLRPs for both cell types to varying degrees. PERI cells supplemented with bBGN or bDCN showed significant or approaching significant increased Young’s modulus, and ultimate tensile strength in PERI cells increased with the addition of 25 nM bBGN. Moreover, tendon proper cell-seeded constructs had increased Young’s modulus for and 25 nM bDCN, and nearly significant increases for 5 nM bBGN and 5 nM bDCN. These results suggest that SLRP supplementation can have positive tenogenic effects on extrinsic PERI cells and intrinsic TP cells.

Cells within a connective tissue can be affected by changes in their tissue niche. For example, if biglycan and decorin expression are absent during development the resulting changes affect the fibril structure with a shift toward larger diameters. In addition to alterations in mechanical properties, such as a failure at lower loads, decreased stiffness, and increase in percent relaxation, knocking out expression of biglycan and decorin affects collagen fiber realignment with a slower response to load [7, 9, 12]. Other knockout SLRP models, including biglycan, decorin, and double biglycan and fibromodulin, have varying degrees of apparent phenotypes including accelerated degeneration of articular cartilage, subchondral sclerosis, reduced growth rate of bone with decreased bone mass, and disruption of proper collagen fibrillogenesis [47–50]. Conversely, supplementation of SLRPs provides evidence for crucial roles in: signaling pathways, such as the *TGF-β* (transforming growth factor beta), *WNT*, *TLR* (toll-like receptor), *EGFR* (epithelial growth factor receptor) internalization, and Akt -dependant/−independent; collagenase shielding; collagen fibrillogenesis in the form of wound healing and scar mitigation; and proteoglycan regulation [26, 50–59].

When evaluating gene expression of the PERI supplemented cells, 5 nM bDCN showed significant increases in *BGN, FMOD,* and *Scleraxis* (*SCX)* and a significant decrease in *CSPG4*. The increase in tendon specific markers may be the result of regulation in the TGFβ pathway with decreased activation of ERK1/2 resulting in increased expression of *SCX* and subsequently SLRPs like *BGN* by TGFβ [60, 61]. Although the cross-linking marker *LOX* tends to decrease, biomechanics (UTS and Young’s Modulus) increase and the fibril distribution is shifted to smaller fibrils indicating that more collagen fibrils are being produced (supported by a trend towards increased *COL1A1* expression) but the fibrils are not maturing and cross-linking remains low (Fig. 5, Fig. S-3). Previous studies in *DCN* knockout mouse models identified an increase in fibril diameter with subsequent decrease in elastic and viscoelastic properties while alterations of the dermatan sulfate side chains had no effect on mechanical properties indicating that the decorin core protein itself is essential for the organization of collagen and its resulting tissue mechanics [12, 26, 47]. In contrast to DCN, the 5 nM bBGN supplementation produced increases in *CSPG4* expression, suggesting that although *BGN* and *DCN* have similar signaling pathways in collagen fibrillogenesis they are antagonistic in perivascularization. In breast carcinoma cells, *DCN* had an anti-angiogenic effect while *BGN* in bone fractures increased pro-angiogenic signals such as *VEGFA* (vascular epithelial growth factor A) showing that *BGN* and *DCN* have antagonistic effects, thus explaining the difference in *CSPG4* expression between bBGN and bDCN in PERI cells [62, 63]. This would indicate that during fibrillogenesis following an injury, *DCN* may play a vital role in mitigating scar formation by preventing vessel growth allowing improved tissue function [64, 65].

Tendon proper and peritenon cells responded differently to supplementation. Such variations could be due to the differences in niche composition within which these cells exist in vivo as well as differences in cell origins, both of which could affect the tenogenic capacity of these cell types [18–20, 66]. The tendon proper niche consists of a stiff, relatively acellular and hypoxic environment with cells undergoing mechanical load and recoil activating characteristic signaling molecules such as *TGFβ* and *Egr1/2* through induction of *SCX* [67]. Other major regulators of tendon maturation and differentiation include *GDF5* (growth and differentiation factor 5) and mohawk (*MKX*) in addition to the SLRPs such as fibromodulin, and biglycan [13, 67, 68]. The peritenon niche is not as clearly defined but is comprised of cells which: (1) express perivascular markers such as endomucin (*EMCN), CD34,* and *CD45*; (2) secrete stimulatory factors during repair; (3) express matrix remodeling-related genes (matrix metalloprotease, *MMP1* and *MMP3,* and *COL3A1*); and (4) possess high cellular phenotypic heterogeneity [18–20, 66].

Peritenon cells had a more pronounced response to SLRP supplementation, particularly for DCN. This suggests that DCN may contribute to the peritenon cells transitioning into a tendon-like phenotype after the initial inflammatory response or that DCN aids in the collagen fibril assembly in the extracellular matrix. These functions could be instrumental for tendon repair since peritenon cells are a highly mobile cell type, reacting immediately in response to an injury. Additionally, from the expression and biomechanics data, DCN supplementation could improve peritenon cell utility in engineered tendon grafts. Though tenocytes (TP cells) might seem to be good tissue engineering candidates, it is interesting to compare the response of PERI cells to SLRP supplementation relative to the non-supplemented TP control. When PERI cell-seeded constructs supplemented with SLRPs were compared to TP control constructs – tenocytes that might be used in grafts. Increases in UTS, Young’s modulus, and MTL were significant or approaching significance with all four doses of SLRPs used (Fig. S-1). Relative to TP control, the constructs with PERI cells had greater collagen content or increased levels approaching significance (Fig. S-1). Moreover, PERI cell-derived constructs had similar matrix assembly marker expression levels (Fig. S-6). Although there were no differences in fibril density or mean diameter, bBGN and bDCN supplemented peritenon cells displayed a shift towards larger fibrils which partially explains the increased UTS, Young’s modulus, and MTL (Fig. S-1, S-4, S-5). The expression of *CSPG4* in bDCN supplemented peritenon constructs was similar control TP cell-derived construct levels, which supports a shift away from a perivascular-like phenotype. This suggests the utility of DCN, in particular for peritenon cells, as a phenotype influencing signaling molecule capable of affecting cells of both regions in the tendon. This finding could have implications during injury repair and cell selection for engineered grafts. Many findings in this study support the supplementation of SLRPs like BGN and DCN in therapeutic strategies. Further studies are required to discern the exact mechanisms by which supplemental SLRPs are affecting PERI cells.

This study has a number of important limitations. First, purified bovine proteins were used with the equine cells instead of equine-derived SLRPs. Second, cellular responses to SLRP supplementation are being described in an in vitro model where the active agent is continuously present in the matrix which would not be the case in vivo in a potentially pathological or inflammatory environment. Third, construct numbers were limited, and thus extensive histological analysis, analyses of other SLRPs, and the combinatorial effects of BGN ad DCN were omitted. Fourth, the tendon cells were isolated from horses with a range of ages and breeds. Fifth, the findings are based upon cells of the equine superficial digital flexor tendon. Therefore, the results might not translate to other tendons and ligaments. Finally, a limited *n* = 5 horses were used in to compare controls and treatments individually to determine if defined and hypothesized improvements in tendon formation were seen with SLRP supplementation. While this might limit the statistical power of the study, it allows for preliminary answers into the efficacy of SLRP supplementation for improving tendon formation. Moving forward, the evaluation of SLRPs in an in vivo injury model could provide further insight into this novel therapeutic intervention for injury repair for equine athletes of all disciplines. Moreover, our findings lend support to further studies that include the incorporation of SLRPs in tendon engineering strategies.

## Supplementary information

**Additional file 1.**

## Data Availability

The datasets used and/or analyzed during the current study are available from the corresponding author on reasonable request.

## Abbreviations

AKT: Protein kinase B signaling pathway
Alpha-MEM: Modified Eagle’s media
BGN: Biglycan
CD34: Cluster of differentiation 34
CD45: Cluster of differentiation 45
COL1A1: Collagen, type 1, alpha 1
COL3A1: Collagen, type 3, alpha 1
CSA: Cross-sectional area
CSPG4: Chondroitin sulfate proteoglycan 4
DCN: Decorin
ECM: Extracellular matrix
EGFR: Epithelial growth factor receptor
EMCN: Endomucin
ERK: Extracellular signal-regulated kinase
FMOD: Fibromodulin
GDF5: Growth and differentiation factor 5
LOX: Lysyl oxidase
MKX: Mohawk
MMP1: Matrix metalloproteinase 1
MMP3: Matrix metalloproteinase 3
MTL: Maximum tensile load
PBS: Phosphate-buffered saline
PERI: Peritenon
POLR2A: RNA polymerase II subunit A
RT-qPCR: Real Time – quantitative polymerase chain reaction
SCX: Scleraxis
SDFT: Superficial digital flexor tendon
SLRP: Small leucine rich proteoglycan
TEM: Transmission electron microscopy
TGF-β: Transforming growth factor β
TLR: Toll-like receptor
TP: Tendon proper
UTS: Ultimate tensile strength
WNT: Wingless and Integrator Complex Subunit 1 signaling
