# Supplementary material for: Adding exogenous biglycan or decorin improves tendon formation for equine peritenon and tendon proper cells in vitro
Source: BMC Musculoskelet Disord. 2020 Sep 23;21:627. doi: 10.1186/s12891-020-03650-2 (PMC7513506; doi:10.1186/s12891-020-03650-2)
Supplement: Supplementary file 1 — Additional file 1. [file 12891_2020_3650_MOESM1_ESM.pdf]

Adding Exogenous Biglycan or Decorin Improves Tendon Formation for Equine Peritenon and Tendon Proper Cells In Vitro

Monica Y. Pechanec[1], Tannah N. Boyd[1], Keith Baar[2,3], Michael J. Mienaltowski[1]

Affiliations:

[1]Department of Animal Science, University of California Davis, 2251 Meyer Hall, One Shields Ave, Davis, CA 95616, USA.

[2]Department of Neurobiology, Physiology, and Behavior, University of California Davis, 195 Briggs Hall, One Shields Ave, Davis, CA 95616, USA.

[3]Department of Physiology and Membrane Biology, University of California Davis School of Medicine, 195 Briggs Hall, One Shields Ave, Davis, CA 95616, USA.

*Corresponding author: mjmienaltowski@ucdavis.edu

Supplementary Information

Table S-1. Equine Taqman primer probe sets designed using Primer3 or predesigned for RT-qPCR.
Figure S-1. Qualitative representation of TEM cross-sections at 5300x magnification.
Figure S-2. Biomechanics comparing tendon proper (TP) control against peritenon cells supplemented with bBGN and bDCN.
Figure S-3. Collagen content and collagen fraction by dry mass comparing TP control and peritenon treatment groups.
Figure S-4. RT-qPCR analysis for TP and peritenon treatment groups.
Figure S-5. Fibril diameter distribution analysis comparing tendon proper control against peritenon samples supplemented with 5 or 25 nM bBGN or bDCN.
Figure S-6. Collagen fibril quantity analysis between TP and peritenon treatment groups.

	Fwd	Rev	Probe
POLR2A	CCAGGATGACCTGACTCACAAA	CGTCGAAGCTGATTGTTGATCT	TGGCGGACATTGTT
BGN	GGTGGGCGTCAACGACTT	GCCATTGTAGTAAGCCCGTTTG	CCCGTGGGTTTCG
DCN	TGCGAAAAGCGGTGTTCA	TGGGTTGGTGCCCAGTTCTA	ACTGAACCAGATGATAGTC
MKX	TCATGTTCCGAAGATGGAGAAA	ATTGTAGCCCCCTTCGTTCA	TCCTCCAAGAAACCAC
COL1A1	GGGCCGAGGGCAACA	GTGGTTTTGTATTCGATCACTGTCTT	CTTCACCTACAGCGTCAC
FMOD	AACCAAGGAGGCCAGACAGA	TGCATTTTGTCTCTCTCAAGTTGAA	ACGTGGTCACTCTGAA
LOX	GCTTGGCCAGCTCAGCAT	TCTTAGCAGCACCCTGTGATCA	CAGGTCAGATGTCAGAGAT
CSPG4	CTCCTGGAGAGAGGTGGAACAG	TCAGTGTCTCGCTCCCATCA	AGCTGATCCGCTATGTG
SCX	ThermoFisher, cat no 4351372, Ec03818452_s1 -- Proprietary information		

Table S-1. Equine Taqman primer probe sets designed using Primer3 or predesigned for RT-qPCR. Forward, reverse, and probe sequences for *POLR2A, BGN, DCN, MKX, COL1A1, FMOD, LOX, CSPG4,* and *SCX* for TaqMan specific RT-qPCR. Primer probe sets underwent dimerization, hairpin formation, melting temperature and GC content scrutiny. All primer probe sets were validated in native equine tendon proper and peritenon tissue.

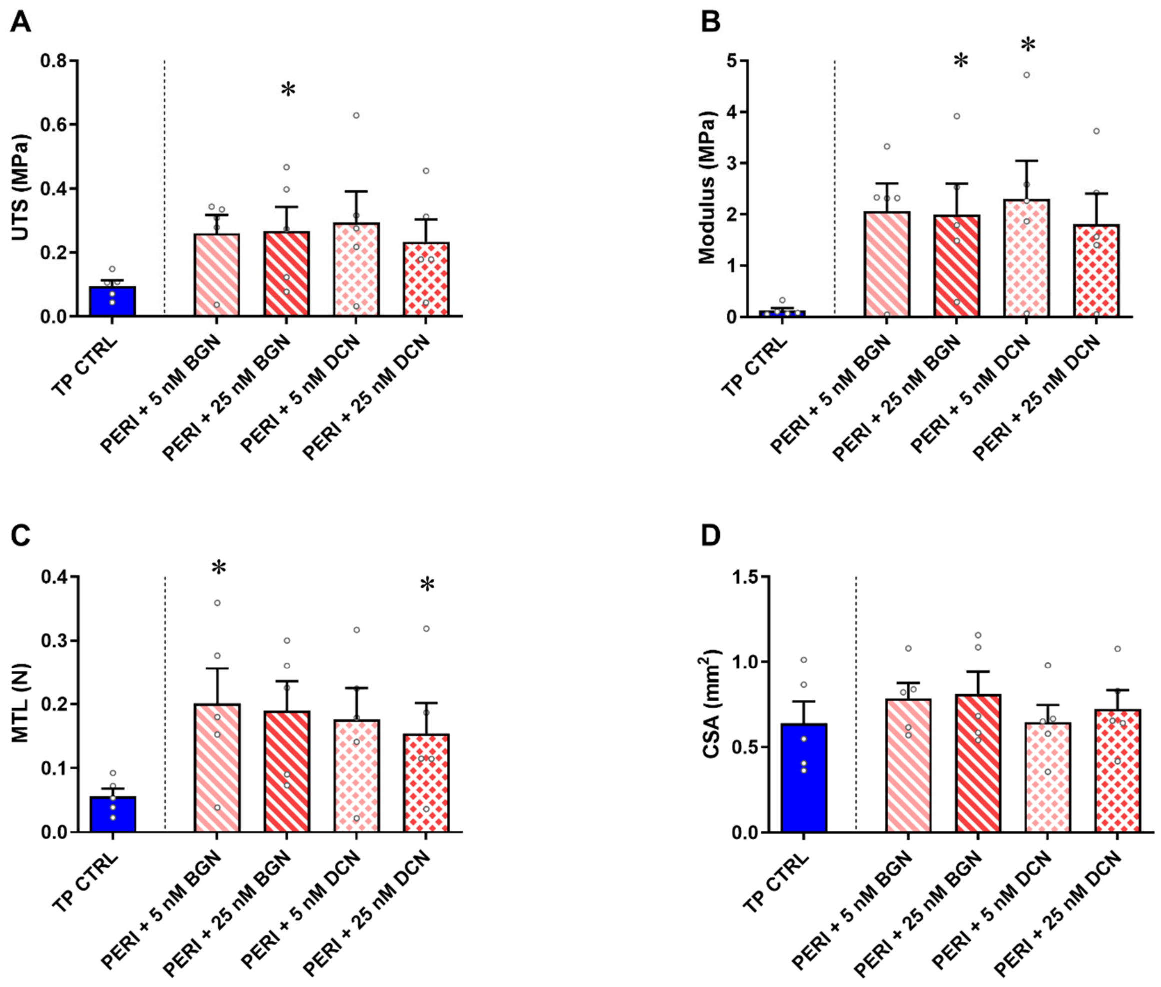

Figure S-1. Biomechanics comparing tendon proper (TP) control against peritenon cells supplemented with bBGN and bDCN. (A) Ultimate tensile strength, **(B)** Young's modulus, and **(C)** maximum tensile load for TP control cells were compared to peritenon treatment groups. TP: tendon proper cells; PERI: peritenon cells; CTRL: no bBGN or bDCN supplementation. Significance is based on one-sided nonparametric Wilcoxon signed-rank tests predicting improvement: *, significant as $p \leq 0.05$, relative to the respective TP or PERI control; n = 5, plotted as mean + SEM. Measurements approaching significance ($p=0.0625$) included: **(A)** 5 nM bBGN, 5 nM bDCN, and 25 nM bDCN; **(B)** 5 nM bBGN and 25 nM bDCN; **(C)** 25 nM bBGN and 5 nM bDCN. Outliers detected by the Grubbs' test in technical replicates (UTS, 2; Young's modulus, 2; MTL, 1; $p < 0.05$) were removed.

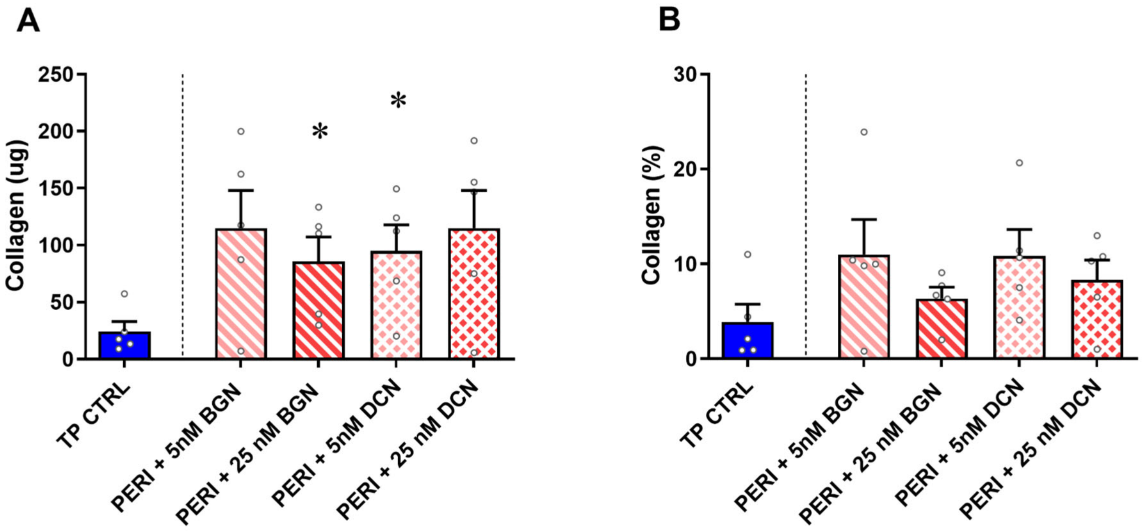

Figure S-2. Collagen content and collagen fraction by dry mass comparing TP control and peritenon treatment groups. Tendon proper (TP) cells were plotted against peritenon treatment groups for **(A)** collagen content and **(B)** collagen fraction by dry mass. TP: tendon proper cells; PERI: peritenon cells; CTRL: no bBGN or bDCN supplementation. Significance is based on one-sided nonparametric Wilcoxon signed-rank tests predicting improvement: *, significant as $p \leq 0.05$, relative to the respective TP or PERI control; n = 5, plotted as mean + SEM. Measurements approaching significance (p=0.0625) included: **(A)** 5 nM bBGN and 25 nM bDCN.

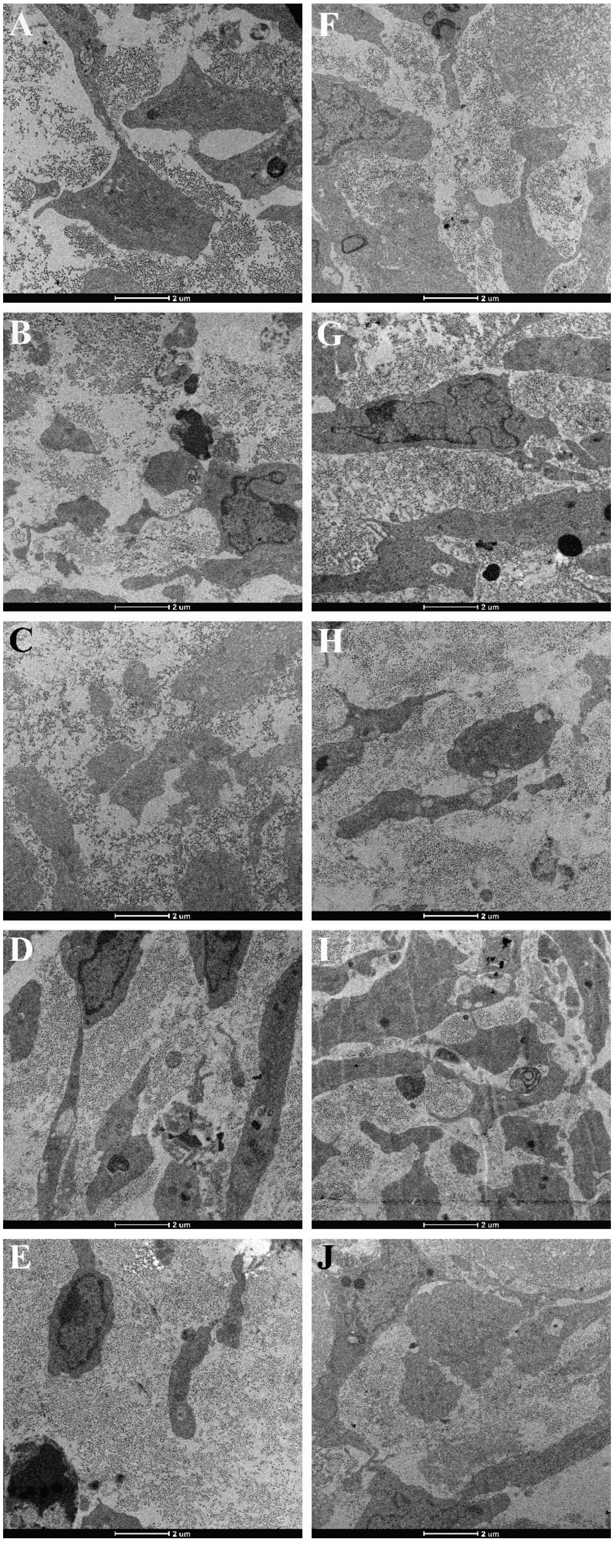
A
F
B
G
C
H
D
I
E
J
2 um

Figure S-3. Qualitative representation of TEM cross-sections at 5300x magnification. **(A)** PERI CTRL, **(B)** PERI + 5 nM bBGN, **(C)** PERI + 25 nM bBGN, **(D)** PERI + 5 nM bDCN, **(E)** PERI + 25 nM bDCN, **(F)** TP CTRL, **(G)** TP + 5 nM bBGN, **(H)** TP + 25 nM bBGN, **(I)** TP + 5 nM bDCN, and **(J)** TP + 25 nM bDCN are represented with low magnification TEM images.

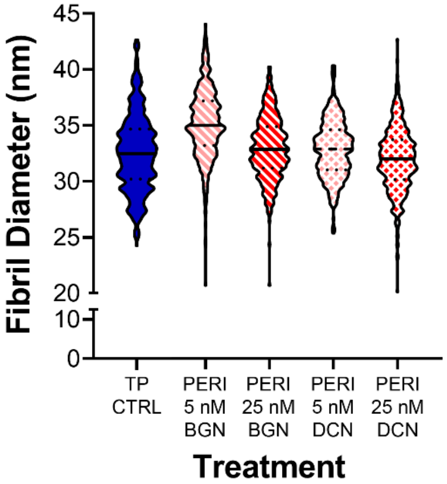

Figure S-4. Fibril diameter distribution analysis comparing tendon proper control against peritenon samples supplemented with 5 or 25 nM bBGN or bDCN. Fibril diameters were calculated and plotted as a violin plot for untreated TP constructs versus PERI +5 nM bBGN, PERI + 25 nM bBGN, PERI + 5 nM bDCN, and PERI + 25 nM, n = 5.

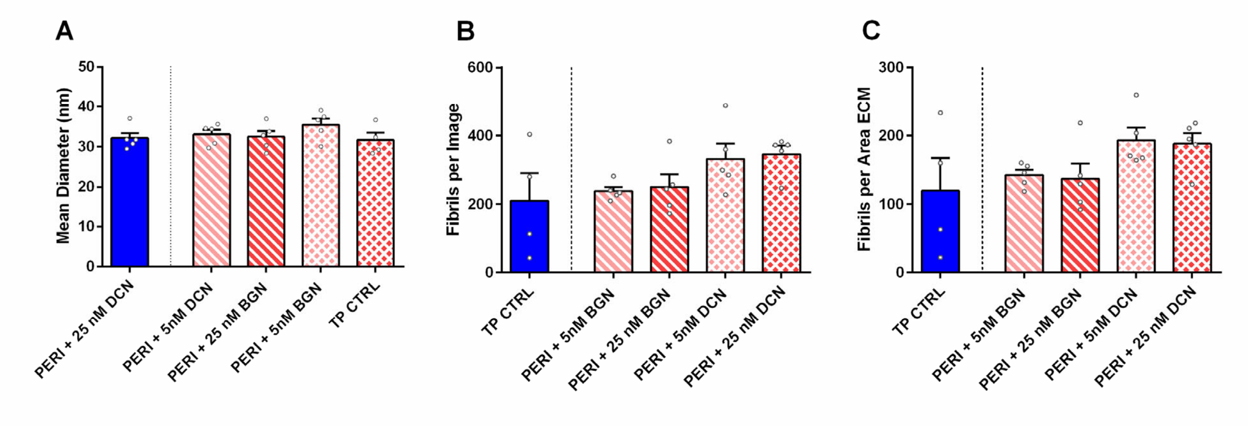

Figure S-5. Collagen fibril quantity analysis between TP and peritenon treatment groups. **(A)** Mean fibril diameter (nm), **(B)** fibril density, and **(C)** fibrils normalized per area of extracellular matrix were compared for TP control and peritenon samples treated with 5 or 25 nM bBGN or bDCN. TP: tendon proper cells; PERI: peritenon cells; CTRL: no bBGN or bDCN supplementation; ECM: extracellular matrix. Significance is based on one-sided nonparametric Wilcoxon signed-rank tests predicting improvement: *, significant as $p \leq 0.05$, relative to the respective TP or PERI control; n = 5.

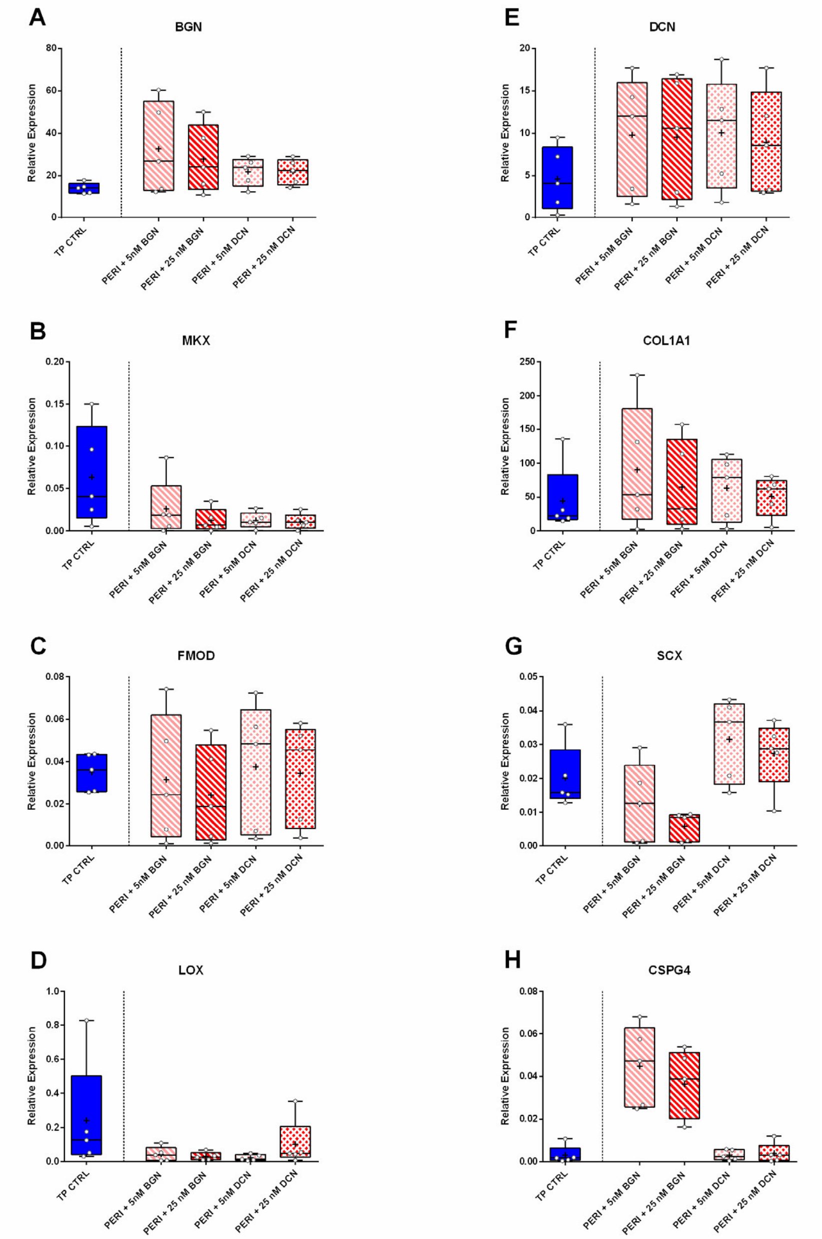

Figure S-6. RT-qPCR analysis for TP and peritenon treatment groups. Gene expression (*POLR2A* was used as the housekeeping gene) for TP control constructs were compared to peritenon constructs supplemented with 5 and 25 nM bBGN or bDCN. TP: tendon proper cells; PERI: peritenon cells; CTRL: no bBGN or bDCN supplementation. Expression is plotted in a box and whisker plot with "+" representing the mean, box representing first-third quartile, line representing median, and whisker representing range. Significance is based on one-sided nonparametric Wilcoxon signed-rank tests

predicting improvement: *, significant as $p \leq 0.05$, relative to the respective TP or PERI control; $n = 5$.
